# Digital ethical reflection in home nursing care: Nurse leaders’ and nurses’ experiences

**DOI:** 10.1177/09697330241244543

**Published:** 2024-04-12

**Authors:** Lena Jakobsen, Rose Mari Olsen, Berit Støre Brinchmann, Siri Andreassen Devik

**Affiliations:** 1786 Nord University; Nord University; Nord University and Nordland Regional Hospital; Nord University

**Keywords:** ethics support, digital ethical reflection, nurse leader, ethics tool, home nursing care, ethics intervention

## Abstract

**Background:**

Nurse leaders increasingly need effective tools that facilitate the prioritisation of ethics and help staff navigate ethical challenges and prevent moral distress. This study examined experiences with a new digital tool for ethical reflection, tailored to improve the capabilities of both leaders and employees in the context of municipal long-term care.

**Aim:**

The aim was to explore the experiences of nurse leaders and nurses in using Digital Ethical Reflection as a tool for ethics work in home nursing care.

**Research design:**

The study employed a qualitative design, incorporating individual and focus group interviews for data collection. Qualitative content analysis was used to analyse the data.

**Participants and research context:**

The participants comprised six nurse leaders and 13 nurses, representing six home care zones across two Norwegian municipalities.

**Ethical considerations:**

The study involved informed, voluntary participation and was approved by the Norwegian Agency for Shared Services in Education and Research.

**Findings:**

Four themes were developed: *a constant walk on the edge between engagement and discouragement* and *lost in translation* describe the process, while *tuning in to the ethical dimension *and *navigating ethical uncertainties* illuminate the experienced significance of Digital Ethical Reflection.

**Conclusion:**

Success with Digital Ethical Reflection in home nursing care depends on clear leadership planning, nurses’ understanding of the tool’s purpose, and active use of digital registrations. Support from ethically interested nurses enhances overall engagement. Further research is needed to explore the potential of Digital Ethical Reflection as an additional tool in long-term care ethics work.

## Introduction

Due to the fundamentals of nursing and caring, healthcare professionals must make ethical decisions and balance moral discomfort when faced with ethical challenges. The psychological harm caused by moral distress can lead to numerous negative consequences for healthcare professionals, including poorer physical, psychological and social wellbeing, and even attrition from the profession.^1^ Given the current and expected future shortage of nurses, recruiting and retaining healthcare professionals is crucial both nationally and globally. Consequently, nurse leaders and healthcare organisations seek to develop tools which proactively address nurses’ perceived ethical challenges in order to prevent uncertainty and potentially moral distress. Such efforts are crucial in maintaining a healthy and ethically sound work environment in healthcare settings. Although digital solutions may contribute to this endeavour, questions remain regarding the types of solutions available and their potential to provide support. This study explores the process and significance of nurse leaders and nurses using a specific digital tool, Digital Ethical Reflection.

## Background

In Norway, municipal healthcare services, including nursing homes and home nursing care, are primarily publicly financed and legally available to all. Each municipality must provide healthcare services to residents of all ages, encompassing substance addiction and psychiatric care as well as residential care. Long-term nursing care includes assisted living facilities and home nursing care in cases where patients are geographically scattered. In recent years, changes in Norwegian healthcare systems, such as earlier hospital discharge and an ageing population with multiple comorbidities, have led to ethically challenging situations and difficult decision-making for healthcare personnel in long-term care.^2,3^ Ethical challenges can arise in numerous caregiving situations, and it is a priority to ensure effective management of these challenges to prevent moral distress. Facilitating the preparation of staff to handle ethical challenges is a crucial aspect of leadership responsibility.^4–6^

Nurse leaders are responsible for leading and managing nurses, as well as ensuring quality patient care.^7^ Research has shown that ethics support for nurses in community health services is enhanced by group reflection, but it must be integrated into everyday practice and supported by both the organisation and the nurse leader.^8,9^

Refining healthcare practices in a dynamic and complex environment characterised by constant changes may impose burdens on nurses, with the introduction of new tasks potentially causing ‘resistance to change’^10^ and ‘change fatigue’.^11^ Nurse leaders often struggle to incorporate changes in the work unit^12^ and systematic ethics work into their daily routines^13^ and seek solutions.^14^

Most ethics interventions aimed at supporting nurses are educational, with less emphasis on identifying ethical issues in professional practice.^15^ Interventions addressing the handling of ethical challenges and thus reducing moral distress are often inadequately evaluated.^1^ Consequently, contextual approaches must be developed to assist nurse leaders in helping nurses navigate the ethical dimension of their work.^16,17^

Due to an ageing population and limited personnel, the Norwegian government has emphasised the need for innovative solutions in healthcare practice.^18,19^ This extends to other Scandinavian primary healthcare contexts, which share similarities and recognise the positive impact of professionals’ engagement in healthcare innovations.^14,20^ Nurse leaders must remain open-minded and interested in using digital solutions, which can facilitate the inclusion of perspectives from both vocal and under-represented nurses, enabling a more comprehensive and systematic approach to addressing ethical issues in the work unit.^14^

Although digital solutions are expected to improve healthcare effectiveness and quality, their potential as managerial tools for nurse leaders, particularly in providing ethics support for nurses, remains largely unexplored. No unified definition exists of what constitutes an ethical tool, with little knowledge regarding the practical workings of ethical tool interventions.^21^ Digital Ethical Reflection was developed to address these gaps and piloted as an ethics support tool among nurses in long-term care in Norway.^22^ The results of this study show that nurses need the involvement of nurse leaders and space for ethical reflection. These results generated an interest in exploring nurse leaders’ expectations regarding the benefits of digital solutions in their ethics work in municipal home nursing care.^14^

### Digital Ethical Reflection

**Figure 1.**
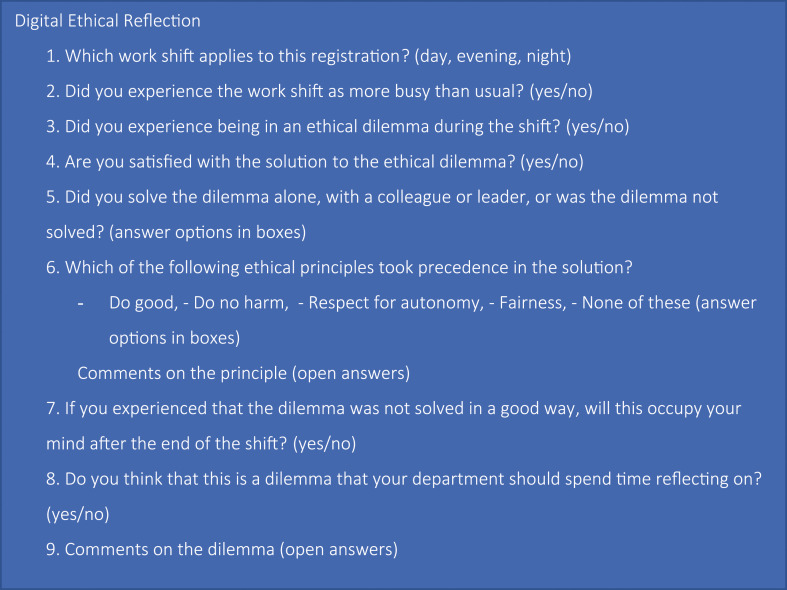
Digital Ethical Reflection questions.

Digital Ethical Reflection (Figure 1) was developed based on nurse leaders’ perception that nurses need to process the ethical challenges they experience. It is a digital registration form, which can be accessed by mobile phone, PC or tablet, providing nurses with a free of cost digital space to document and describe ethical challenges encountered during their shifts.^22^ The form comprises nine queries (Figure 1), aligning with the four central prima facie principles of bioethics as described by Beauchamp and Childress.^23^ The pilot phase, conducted with nurses in two long-term care units across two municipalities, revealed the significance of nurse leaders actively reviewing the registrations created in Digital Ethical Reflection.^22^ A separate investigation was then initiated in two new municipalities that were not involved in the pilot phase. The nurse leaders who were recruited in the new municipalities shared expectations and input on how Digital Ethical Reflection could be used as a future management tool for ethics work among their employees. Nurse leaders’ expectations and plan for using the tool have been published.^12^

The present article describes the practical testing of the tool, during a period ranging from two to seven months, and the experiences of the nurse leaders and their nursing staff. The participants in present study and in previous study^12^ are not identical to participants in the pilot phase.^20^

Digital Ethical Reflection stores registrations securely and anonymously in a database hosted by the University of Oslo, Norway. These registrations were converted to simple statistics and shared with each nurse leader monthly during the study. The nurse leader was free to use this information in any way deemed appropriate, including ethical reflection groups.

### Aim and research questions

This research study aimed to explore nurse leaders’ and nurses’ experiences using Digital Ethical Reflection as a tool to lead ethics work in home nursing care. It intended to explore experiences related to process and significance, represented by two research questions: (1) What was experienced positively and what was challenging when participating in Digital Ethical Reflection? and (2) What significance did Digital Ethical Reflection have for leading and participating in ethics work?

## Methods

### Study design

This research employed a qualitative design, using content analysis to analyse the empirical data. The qualitative approach aims to elucidate rich descriptions derived from human experiences regarding a specific phenomenon. This approach is rooted in the recognition of the existence of multiple realities and seeks to uncover the intricate interplay between researchers and participants, emphasising contextual and value-bond perspectives.^24^ Furthermore, we explored the utility of a specific ethics tool (Digital Ethical Reflection) in everyday practice.^25^

### Recruitment

An initial inquiry was made via email to the ethics consultant at The Norwegian Association of Local and Regional Authorities (KS). This inquiry allowed contact information to be sent to managers in four municipalities. The study information was then distributed to all nurse leaders in nursing homes and home care nursing in their department. This approach recruited nine nurse leaders in one municipality from home nursing care services willing to participate in the study. Snowball recruitment generated one additional nurse leader participant from another municipality. The nurse leaders who consented to participate assisted in passing on invitations to their employees (nurses) to participate in focus group interviews towards the end of the project period (Table 2).

### Participants and study setting

The study was conducted in two municipalities in Norway between autumn 2021 and spring 2023. Nurse leaders from four home nursing care (HNC) units and two home nursing care units for substance addiction and psychiatric care (HNC-SAP; Table 1) signed up to participate. The nurse leaders managed between 14 and 65 nursing staff within their work unit. The same nurse leaders had participated in a study exploring their expectations of Digital Ethical Reflection.^14^ Due to sick leave and workplace transitions, the final count of participating nurse leaders remained at six, plus the nurses (who consented to participate in focus group interviews) in their respective work units, who had various qualifications (Table 2). The total number of participants was 19: three men and 16 women.

**Table 1.** Characteristics of nurse leaders.

Workplace	Age in years	Leader education	Years as nurse leader
Home nursing care (HNC)	42	Yes	3
Home nursing care (HNC)	45	Yes	7
Home nursing care (HNC)	40	No	1
Home nursing care (HNC)	31	No	5
Home nursing care for substance addiction and psychiatric care (HNC-SAP)	54	No	2
Home nursing care for substance addiction and psychiatric care (HNC-SAP)	46	Yes	6

**Table 2.** Characteristics of nurses and nurse leaders in focus groups.

	Total	Focus group 1	Focus group 2	Focus group 3	Focus group 4
Participants	19	6	4	3	6
Age in years (mean)		30–65 (52.8)	31–50 (41)	30–62 (41.3)	31–56 (43)
Registered nurse/disability nurse	6	3	2	1	
Assistant nurse^a^	2	1	1		
Enrolled nurse^b^	5	2	1	2	
Nurse leader					6
Home nursing care (HNC)	4		2	2	4
Home nursing care for substance addiction and psychiatric care (HNC-SAP)	9	6	2	1	2

^a^Personnel without formal health education.

^b^Enrolled nurse has two years of upper secondary healthcare education and two years of experience as apprentices before obtaining a professional certificate.

The nurse leaders were acquainted with the digital tool beforehand and had actively engaged in workshops that covered both the anticipated outcomes of the tool and the necessary adjustments.^12^ Subsequently, the testing of the tool was scheduled to occur among their respective employees. During departmental meetings, the nurse leaders themselves communicated information about the tool to the nurses. To facilitate this information dissemination, the first author created written materials, including leaflets and posters.

### Data collection

**Figure 2.**
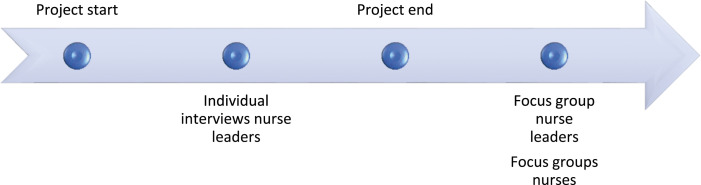
Data collection timeline.

Data were collected at two time points: (a) individual interviews with nurse leaders two months after the study began and (b) focus groups with nurses and nurse leaders (in separate groups) after the study ended (Figure 2). Collecting data from multiple stakeholders and time points enriched our comprehension of the participants’ concerns and viewpoints. We sought to capture both their experiences using Digital Ethical Reflection and its perceived significance.

### Interviews

Both individual interviews and focus groups were conducted. The individual interviews were held digitally in an informal setting between the researcher LJ and the respective nurse leader. The initial question posed to each nurse leader was, ‘How is the Digital Ethical Reflection initiative progressing?’ The conversations, lasting approximately 20–30 min, were recorded and transcribed verbatim by LJ. Employing individual interviews at this stage of the study served a twofold purpose. First, it provided a means to gauge the progress and effectiveness of the Digital Ethical Reflection implementation. Second, it facilitated the identification of potential barriers encountered by nurse leaders in the process, enabling a detailed discussion of the necessary remedial actions. The use of individual interviews ensured a comprehensive understanding of the challenges faced by nurse leaders.

The focus groups with nurse leaders were conducted digital using Teams telephony. The discussions began by asking ‘How did you experience using Digital Ethical Reflection?’ and ‘Can you give an example of ethical challenges from the digital registrations?’ Follow-up questions such as ‘Can you elaborate further?’ and ‘Can you give an example?’ were asked if the answers were unclear. Explicit questions to nurse leaders were ‘How did you use the data from the registrations?’ and for nurses ‘How did your nurse leader use the registrations in your work unit?’ Focus groups were chosen because they yield a synergetic effect due to the communication between participants, who share experiences and cultural presuppositions that are fruitful sources of data.^26^

At the end of the study period, which varied between two and seven months in the different work units, the final data were collected from four focus groups (Table 2). The nurse leaders knew each other through work. The focus groups with nurses took place as physical meetings at a neutral town hall meeting room. Some nurses worked together; others had never met. All focus groups were moderated by LJ and co-moderated by SAD.

### Data analysis

Texts were analysed using inductive content analysis.^27,28^ The unit of analysis was the transcribed interview texts from all interviews (individual interviews with nurse leaders, focus groups with nurse leaders, nurse focus groups, and observational notes from interviews and informal meetings). The stated research questions (see Aim) were used in the analysis to identify the participants’ experiences of the process and the perceived importance (significance) of using Digital Ethical Reflection. It began with all researchers familiarising themselves with the text by reading and re-reading each interview separately to grasp the whole and identify meaningful units in the texts. All researchers met and discussed imprints of the texts before LJ organised the codes into fitting sub-themes using NVivo 14 software. After the initial coding and categorisation, all researchers met and discussed the latent meanings of the codes and categorisation and agreed on preliminary themes.

The abstraction and interpretation of the texts were approached with an evaluative focus: identifying, coding, categorising, and producing themes. Although the coding of meaning units was kept manifest, the interpretation level increased during the categorisation and theme-producing phase.^28^ All researchers had prior experience in qualitative research and the context of nursing. We were aware of the presuppositions inevitable in qualitative research and had related discussions during the analysis process to resolve disagreements in the interpretation of categories and themes.

### Study trustworthiness

To enhance the trustworthiness and rigour of our qualitative research, we adhere to the standards as described by Lincoln and Guba.^24^ The context and the participants are described in as much detail as confidentiality considerations allow to ensure transparency. To capture the core themes within the data text, we selected segments of the data that were either frequently echoed across participants’ responses or were specifically emphasised by the participants themselves. The connection between the empirical data and our findings is strengthened through direct quotations from participants’ responses. Additionally, the ongoing contact between first author and the participating nurse leaders serve as an example of ‘prolonged engagement’, which aids in the study’s development process and ultimately enriches the results. Each researcher brings a background in qualitative research and an understanding of the nursing context. Recognising that bringing certain preconceptions to qualitative research is unavoidable, we actively engaged in team discussions throughout the research process – from planning and project execution to interviewing, data analysis, and the presentation of our findings.

### Ethical considerations

This study was approved by the Norwegian Agency for Shared Services in Education and Research, project number 400394. All participation was voluntary, and written informed consent was obtained before the interviews. The nurses voluntarily used the tool, and the registrations they made were not classified as research data. Instead, they were intended for use in the department’s ethics work, and the nurse leaders received them as anonymised information. However, nurse leaders assumed a dual role in this study, which obliged them to fulfil their usual responsibilities as professional managers to promote competence and ethics among their staff, while also acting as research participants to facilitate optimal data collection. Despite the project’s emphasis on voluntary registrations, the managers’ dual role may have inadvertently exerted additional pressure on nurses’ commitment.

## Findings

The analysis ended with four main themes and nine sub-themes reflecting the leaders’ and nurses’ experiences (Table 3). The themes *a constant walk on the edge between engagement and discouragement* and *lost in translation* encapsulate the dynamics of the process. The themes *tuning in to the ethical dimension* and *navigating ethical uncertainties* shed light on the participants’ experiences regarding the significance of Digital Ethical Reflection.

**Table 3.** Example of data interpretation.

Condensed meaning unit	Code	Sub-theme	Theme
It was a chance to learn more about what staff were thinking (nurse leader)	Learning opportunity	*Leadership influence*	A constant walk on the edge between engagement and discouragement
If the nurse leader constantly reminded, it was remembered to do it (nurse)	Needed to be reminded		
Not much focus on it after a while (nurse leader)	Decreasing interest	*Engagement ambivalence*	
Sparking an interest in ethics work (nurse)	Increasing interest		
If organised, there was enough time to do it (nurse)	Effective organisational skills	*Commitment to organise for reflective practice*	
Given additional time for doing it during work hours (nurse leader)	Allocated time		
Thought the registration was for the benefit of the research team (nurse)	Missing the purpose	*Limited clarity*	Lost in translation
Did not obtain enough information about it (nurse)	Clueless		
Thought it was only about patients (nurse)	Unclear instructions	*Misunderstandings*	
Need to avoid being on autopilot and not think (nurse)	Examining old habits	*Avoid autopiloting*	Tuning in to the ethical dimension
Asking oneself questions about choices made (nurse)	Self-reflection		
Shared and discussed the registrations with colleagues (nurse)	Discussing	*Promoting collaboration and participation*	
Helped that others acknowledged the same challenges (nurse leader)	Sharing experiences		
It was difficult sometimes finding the right category or not knowing what an ethical challenge is (nurse)	Inadequate ethical knowledge	*Ethical uncertainties*	Navigating ethical uncertainties
Insecure in own ethical decision-making skills or need feedback and an ‘answer’ (nurse)	Insecure		
Threshold for making registrations of dilemmas if unexperienced (nurse)	Inexperienced	*Ethical terminology*	
Felt empowered to advocate for patients because learned ethical language (nurse)	Empowered		

### A constant walk on the edge between engagement and discouragement

This theme captures the delicate balance between engagement and resistance. Participants, including nurses and nurse leaders, exhibited a wide range of attitudes, ranging from enthusiasm and a desire for learning and facilitating participation to indifference, reluctance, and even resistance.

In practice, this meant that the nurse leader was considered responsible for reminding the nurses to register and providing the link to the registration.

Nurse leaders often grappled with managing participation in the study and maintaining consistent motivation among nurses. Some nurse leaders eventually abandoned their efforts, feeling torn between demanding participation and asking for it: ‘I do feel like I’ve pushed a lot. So, I’ve actually given up because I can’t keep nagging my employees. I think I’ve tried… I’ve pushed as much as I can’ (FG 4, HNC).

Participants emphasised the importance of nurses being willing to engage but faced challenges, such as postponing, prioritising other tasks, and forgetting to complete registrations. Several nurses talked about reflection as a ‘luxury’ they lacked time for:

Well, as we’ve said, we have a more demanding job than many others. We have a lot of extra work due to things like sick leave, short staffing, and new patients – many little things that we handle alongside. And you don’t really have time for doing reflection, it’s like extra luxury, you know (FG 3, HNC).

Many nurses greeted the study with reluctance and weariness: ‘I was thinking to myself when the nurse leader introduced it to us, “Here we go again – another application we need to make registrations…”’ (FG 2, HNC). One nurse leader expressed frustration when the nurses chose not to participate since it was not mandatory.

The heavy workload and task-oriented focus of nurses in home nursing care posed challenges in finding time for registrations. Nevertheless, some nurses managed to integrate digital reflection into their workflow by adjusting and prioritising time. Approaches varied on how to use the registrations in the different work units. One nurse leader advocated for additional space and time for ethical reflection meetings:

When you start with something that’s interesting, you need time for it. It’s not useful to sit down for just 15 minutes and ask: what has the dilemma been today? You need time to delve into it (FG 4, HNC-SAP).

Other nurse leaders and nurses found more value in ad hoc discussions during breaks and patient reports:

We are very present, right? We’re part of daily reports, we join in for lunches, and we engage in professional discussions. I find that more exciting than a simple ‘Have you experienced this, yes/no’ and maybe three words in a free-text that doesn’t really tell me much. I get more from being in meetings with my nurse colleagues (FG 4, HNC).

We bring up ethical issues almost every day with our nurse leader who’s present during reports. She needs to be there to hear what’s been done and what hasn’t, and so on (FG 3, HNC).

The findings demonstrated varying levels of commitment among both nurse leaders and nurses, influenced by their roles and attitudes, in terms of their willingness to participate in and allocate time to the reflection work.

### Lost in translation

Some nurses felt their nurse leader did not provide enough information about the study. The intention was unclear, and they were not told that registrations could serve as a starting point in an ethical reflection group, for example:

I feel that it’s a bit sad that we were asked to participate in that study, but we didn’t really get enough information about it… ’Cause this is pretty important stuff. Someone could have taken a little time to tell us more about it. Maybe then people would’ve been more engaged and willing to join in (FG 3, HNC).

Other nurses expressed that the nurse leader had provided poor information, leading to missed opportunities for registering ethical challenges: ‘I had no idea that I could register my experiences with colleagues; I thought it was all about the patients. If I had known, I would have registered a lot more’ (FG 1, HNC-SAP). This led to missed opportunities for registering ethical challenges because some were related to colleagues and their unethical behaviours.

Most of the nurse leaders claimed they had communicated sufficiently with clear information about the purpose of the study: ‘I probably have some employees who haven’t been interested… or they have probably been interested but maybe haven’t understood the study, regardless of how good the information has been’ (FG 4, HNC).

In addition to confusion regarding the study aim, one nurse leader expressed concern about language barriers:

I’ve sent out information about the study and brought it up during staff meetings… but I must admit, there are many who don’t want to participate. Then I went directly to nurses I thought would have been positive, but they were not. I was a bit surprised by that! I am not sure if it’s because they have poor Norwegian language knowledge... (nurse leader, HNC, individual interview).

These findings underscore how the nurses felt a need for effective and comprehensive information-sharing and the nurse leaders wanted to ensure the nurses’ engagement and understanding.

### Tuning in to the ethical dimension

This theme emphasises how personal reflection using Digital Ethical Reflection can support collaborative deliberation and be part of a reflection process loop, starting with nurses’ introspection and proceeding to a mutual deliberation of ethical challenges.

For many of the nurses, the registrations served as a reminder of the ethical aspects of their practice. They also instilled an awareness of the number of ethical decisions they made throughout the day and made them question their choices:

For me, doing the registrations gave me aha moments… ‘Oh wow, what am I doing here?’ When you find yourself in difficult situations and you have to make decisions on your own, it’s clear that you have many thoughts afterward… or at least one: ‘Did I make a mistake, should I have done it differently?’ (FG 1, HNC-SAP).

For some nurses, Digital Ethical Reflection opened discussions around ethical issues in the work unit. Writing in the open-text spaces in Digital Ethical Reflection also contributed to the reflection process:

Sitting down to write about the ethical challenge is asking one to think it through in a slightly different way. It is kind of structuring the whole experience. I personally thought it was nice, as it was also nice that we addressed it in some of the reflection meetings we had (FG 2, HNC-SAP).

In the reflection groups where the nurse leader used anonymised examples from the registrations, the staff identified with the ethical challenges and were gratified to know that colleagues experienced the same ones:

I feel doing these registrations were really a good thing, it made us realise that there were many of our colleagues which were experiencing the same challenges. A lot of people recognised what was written… And several times somebody raised their hand and said, ‘I think I wrote about that!’ (FG 2, HNC-SAP).

Some nurse leaders structured ethical deliberations after receiving the digital ethical monthly report. This approach fostered supportive relationships and collaborative decision-making within the work unit, positively impacting the organisational culture:

Ethics is something everybody knows something about. Having that space where you can bring forth what you carry, where there’s no right or wrong… It’s been a safe space for people to address things that might have been difficult. (FG 4, HNC-SAP)

These findings depict the participants’ experiences with Digital Ethical Reflection as promoting collaboration and reflective practice.

### Navigating ethical uncertainties

This theme highlights the ambiguity surrounding ethical challenges and principles, as well as nurses’ need for guidance in handling complex ethical situations.

Both nurses and nurse leaders worried that nurses might be unsure what constitutes an ethical challenge: ‘When I reviewed the answers in the report, I realised that there was much confusion on what an ethical challenge or an ethical dilemma actually is’ (nurse leader, HNC). At the start of the study, the nurse leaders emphasised to the nurses that they weren’t required to know ethical principles; they should just register anything that made them uncomfortable:

I told my staff to not be concerned about whether it’s an ethical dilemma or not, because in my experience when it comes to ethical reflection, no one dares to bring up a topic. They’re so afraid that everyone will think they’re way off track that it’s not an ethical dilemma in any way (FG 4, HNC).

Many nurses found placing their ethical challenge within a specific ethical principle difficult, leading them to use the open comments feature to provide additional explanations about what caused them discomfort.

I think I wrote additional explanations every time I registered a dilemma, except one time. When I wrote about it, I reflected about it once more, and it made me surer if it was a good decision or if I needed to do something different (FG 2, HNC-SAP).

Some nurses grew familiar with ethical principles using Digital Ethical Reflection, empowering them to advocate for patients: ‘When you get used to the ethical concepts and get to know them more, then you dare to participate in the discussions on behalf of the patient’ (FG 3, HNC).

Many nurses were disappointed at the lack of feedback from the nurse leader on the registrations. The opportunity to learn from mistakes or receive approval for how they handled a specific ethical challenge was considered important for most nurses, whether or not they found Digital Ethical Reflection useful:

After a challenging situation, I often wonder if my decision was right. Hearing about the challenges others have met is helpful for me if I should find myself in a similar situation… then I know how to proceed, but when you finish writing the Digital Ethical Reflection, and everything seems to be cast aside, you’re left wondering if what you’re doing is the right and responsible way (FG 3, HNC).

These findings highlight the nurses’ expressed need for guidance and support in navigating ethical complexity.

## Discussion

Our intention with this study was to examine the process and potential significance of Digital Ethical Reflection as a tool for nurse leaders’ ethics work in long-term care. The themes *a constant walk on the edge between engagement and discouragement* and *lost in translation* encapsulate the dynamics of the study process. The themes *tuning in to the ethical dimension* and *navigating ethical uncertainties* shed light on the participants’ experiences regarding the significance of Digital Ethical Reflection. The findings reflect the varying experiences of the participants, encompassing the perspectives of both nurses and nurse leaders, and demonstrate the diverse experiences related to systematic ethics work in long-term care.

Nurses opposing participation or being uninterested came as a surprise to the nurse leaders. Nurse leaders must grasp the strategic mechanisms required to effectively mobilise and engage their nurses, given the inevitability of novel routines, reorganisation, and advancements in patient care.^29^ The reluctance to prioritise time for participation aligns with what is described in the literature as ‘change resistance’, which is likely due to interconnected individual, interpersonal, and organisational factors.^10^ In our study, the quality of information given by nurse leaders was considered low by the nurses. Resistance to change increases when nurses feel uninvolved from the outset or when the perceived usefulness and professional benefits remain unclear.^30^ Nurse leaders who provided extended feedback on the registrations might have engendered a sense of utility regarding digital reflection, potentially bolstering participation. This finding is sustained by prior research suggesting a direct link between perceived usefulness and resistance to change.^31^

Another concept influencing nurses’ engagement could derive from fatigue caused by frequent organisational restructurings and task alterations. Research describes ‘change fatigue’ as ‘the repercussion of attempting to keep up with evidence-based practice due to the institution’s implementation strategies and timing without consideration of nursing workload’.^11^ Heavy workload and time restrictions were expressed as deterrents to participating in our study and are also considered inhibitors of positive change behaviour.^32^ One way of preventing work fatigue might be for nurses to limit their work effort and engagement.^33^ The indifferent attitude shown by some nurses when the nurse leader attempted to implement the study might be considered a red flag. Investigating the reasons for the nurses’ disinterest could have led the nurse leader to an understanding of the experience of a lack of information or purpose and led to an improved strategy for integrating the study into the nurses’ daily work.

Patel et al. (2022) concluded that ‘nurses need to go beyond reflection-on-action and also include reflection-in-action and reflection-for-action as part of their practice’.^34^ Reflecting on the causes of discomfort can be demanding because it requires a deep examination of one’s conduct and choices. The self-reflection process depends on a certain amount of peace and time, as does moral deliberation in groups. The workplace context was significant to the time and opportunity available for ethics work. The opportunity that the digital tool gave for ethical reflection, personal or in groups, was more likely to be facilitated in the context of assisted living facilities for substance abuse and psychiatric care than in other home nursing care contexts.

Digital Ethical Reflection might reduce the gap between ethical principles and nursing practice, functioning as a catalyst to stimulate personal reflection and foster discourse among colleagues. In this sense, technology can advance nursing knowledge and professional development.^35^ Although offering diverse approaches to ethics work is valuable, the most salient objective is facilitating collaborative deliberation among nurses and their nurse leader. After all, developing a common morality is done in unity.^36^

Dedicated ethical reflection may appear daunting and subject to competing priorities. However, our study, along with existing research, underscores the value of ethics work originating from the practical experiences of ethical challenges that nurses face in their daily work.^8,9^

### Strengths and limitations

Despite the modest participant count, a strength of our study was diversifying its participants across various home nursing care settings. Further, the findings are supported by quotes from the participants, which could help make the findings transferable. A study limitation was the fluctuating work situations of nurse leaders, such as changing workplaces, taking sick leave, and resigning from their positions. These circumstances led to participant dropouts which reduced the number of participants.

Critics might argue that the current study adopted a ‘top-down’ approach. In this scenario, management presents improvement measures, potentially creating a perception of imposition on employees. In contrast, successful implementation processes are more likely when employees actively participate, take ownership of problems and solutions, and play a substantial role in quality improvements, commonly known as a ‘bottom-up’ approach.^37^ This distinction is crucial for considering the broader application of the study’s results.

### Implications

This study offers valuable insights into the complex processes involved in healthcare interventions involving nurse leaders and nurses. Using a digital solution for registration and reflection enhances the documentation of ethical challenges from nurses to nurse leaders and increases nurses’ ethical competencies. This not only enables learning from past experiences but also furnishes guidance for addressing ethical challenges when used as a catalyst for ethical deliberation in the work unit. Understanding how nurse leaders and nurses perceive Digital Ethical Reflection as a managerial ethics tool can inform the development and implementation of such tools in the future.

## Conclusion

The success of Digital Ethical Reflection seems to depend on the nurse leader having a clear and strategic plan for how to use the tool so that nurses understand the aim of the registrations. The leader must allocate sufficient time for reflection and actively use the ethical challenges gathered from nurses’ registrations. Additionally, seeking support from nurses with a specific interest in ethics can enhance overall engagement. However, a one-size-fits-all approach to ethics is inadequate, making the Digital Ethical Reflection a potential additional tool for ethics work in long-term care. Further research is necessary to examine how digital tools can facilitate ethics work in long-term care.
